# Patient Controlled Epidural Analgesia during Labour: Effect of Addition of Background Infusion on Quality of Analgesia & Maternal Satisfaction

**Published:** 2009-12

**Authors:** Uma Srivastava, Amrita Gupta, Surekha Saxena, Aditya Kumar, Saroj Singh, Namita Saraswat, Abhijeet R Mishra, Ashish Kannaujia, Sukhdev Mishra

**Affiliations:** 1 Professor, Research scholar Agra University; 2 PG Residents, Deptt of Anaesthesiology and Critical Care, Research scholar Agra University; 3 Professor, Research scholar Agra University; 4 Professor, Research scholar Agra University; 5 Professor, Deptt of Obs & Gynae SN Medical College Agra; 6 PG Residents, Deptt of Anaesthesiology and Critical Care, Research scholar Agra University; 7 PG Residents, Deptt of Anaesthesiology and Critical Care, Research scholar Agra University; 8 Lecturer, Research scholar Agra University; 9 M.Stat, PhD Biostatistics , Research scholar Agra University

**Keywords:** PCEA, Background infusion, Labor analgesia

## Abstract

Patient controlled epidural analgesia (PCEA) is a well established technique for pain relief during labor. But the inclusion of continuous background infusion to PCEA is controversial. The aim of this study was to assess whether the use of continuous infusion along with PCEA was beneficial for laboring women with regards to quality of analgesia, maternal satisfaction and neonatal outcome in comparison to PCEA alone. Fifty five parturients received epidural bolus of 10ml solution containing 0.125% bupivacaine +2 µg.ml^−1^ of fentanyl. For maintenance of analgesia the patients of Group PCEA self administered 8 ml bolus with lockout interval of 20 minutes of above solution on demand with no basal infusion. While the patients of Group PCEA + CI received continuous epidural infusion at the rate of 10 ml.hr-1 along with self administered boluses of 3 ml with lockout interval of 10 minutes of similar epidural solution. Patients of both groups were given rescue boluses by the anaesthetists for distressing pain. Verbal analogue pain scores, incidence of distressing pain, need of supplementary/rescue boluses, dose of bupivacaine consumed, maternal satisfaction and neonatal Apgar scores were recorded. No significant difference was observed between mean VAS pain scores during labor, maternal satisfaction, mode of delivery or neonatal Apgar scores. But more patients (n=8) required rescue boluses in PCEA group for distressing pain. The total volume consumed of bupivacaine and opioid was slightly more in PCEA + CI group. In both the techniques the highest sensory level, degree of motor block were comparable & prolongation of labor was not seen. It was concluded that both the techniques provided equivalent labor analgesia, maternal satisfaction and neonatal Apgar scores. PCEA along with continuous infusion at the rate of 10 ml/ hr resulted in lesser incidence of distressing pain and need for rescue analgesic. Although this group consumed higher dose of bupivacaine, it did not affect maternal or neonatal safety.

## Introduction

Safe foetal outcome without any adverse maternal effect is the chief goal of pain relief during labor and lumbar epidural analgesia is the most efficient and widely employed modality for this. After initiation of epidural analgesia by bolus dose, many techniques have evolved for subsequent maintenance of analgesia such as intermittent boluses by the clinicians, midwives or patient herself and continuous epidural infusion. The concept of patient controlled epidural analgesia (PCEA) was introduced during labor so that laboring women could control the dose of epidural medication according to their need due to change in labor and pain patterns^1^,^2^. PCEA is now established and well accepted technique both by parturients as well as obstetricians^3^. But the role of continuous background infusion to PCEA is still a debated issue^4^–^8^.

The purpose of this prospective randomized study was to investigate whether addition of continuous basal infusion to PCEA affected quality of labor analgesia maternal satisfaction, mode of delivery and neonatal outcome. We also investigated the need for anaesthesiologist administered additional rescue topups and the dose of bupivacaine consumed during labor & vaginal delivery.

## Methods

The study was conducted on 55 full term parturient women of ASA status I and II of mixed parity who were willing for epidural analgesia during labor. The women included in the study had singleton pregnancy with vertex presentation, had no contraindication to epidural analgesia and who were able to use PCA pump. The study was undertaken after Institutional Ethical Committee approval and informed written consent.

Before institution of the epidural block demo- graphic data, parity, gestational age condition of the membrane, cervical dilatation, vital parameters and FHR were noted and 500 ml of Ringer lactate was given intravenously. Epidural block was given after 3-4 cm cervical dilatation in sitting or lateral position by a third year anaesthesia resident in L_2-4_ interspace with 18 G Touhy's needle. Loss of resistance to air was used for localization of epidural space. The catheter was taped in place, leaving 3-4 cm in the space. The patients were randomly divided into two groups according to computer generated randomization.

All the patients received an initial bolus dose of a 10 ml solution containing 0.125% bupivacaine + 2 mcg.ml^−1^ of fentanyl through the epidural catheter. The anaesthetist programmed the PCEA pump (Fresenius vial SA, Le Grand Chemin, 38590 Brezins France) to deliver 8 ml bolus of the above solution on demand with lockout interval of 20 minutes (Group PCEA, n = 30) or to deliver a continuous epidural infusion of above solution at the rate of 10 ml/hr with demand bolus of 3 ml of above solution with lockout interval of 10 minutes (Group PCEA + C I). In both the groups maximum upper limit of epidural solution was 25ml/ hour. The women were told to press the demand button whenever the analgesia was inadequate and to expect some relief within 5–6 minutes. The anaesthetist was called to give additional epidural injection if analgesia was unsatisfactory and pain was distressing. The number of times the anaesthetist gave rescue analgesic was recorded.

Pain relief was assessed at 20 minutes and then hourly by an observer (who was unaware of the group) using Verbal Analog Scale of 0 – 10 where 0= no pain & 10= worst pain. Highest level of sensory block, degree of motor block (Bromage Scale), number of additional supplements by anaesthetist, total dose of bupivacaine consumed per hour & and during the labor, mode of delivery and neonatal Apgar scores were recorded. SpO2, heart rate and BP were monitored throughout the labor. FHR was monitored continuously using cardiotochograph. Any adverse effect such as weakness of limbs, hypotension, arterial desaturation and pruritus were noted and managed if required.

For statistical calculation the software SAS 9.13 was used. The data are presented as mean ± SD or % or number of patients. One way analysis of variance was used to evaluate differences in continuous variables across the groups followed by Bonferroni's test for multiple comparisons. X^2^ test was used to compare analgesic quality and p<0.05 was considered as significant. We calculated that a sample size of minimum 25 patients per group would give the study a power of 80% to detect a statistically significant difference in the verbal analogue pain score.

## Results

There was no difference between the two groups regarding maternal demography and obstetrical characteristics. Duration of labor, incidence of caesarean delivery and Apgar scores were also comparable between the groups (Table 1). Total volume of bupivacaine used during labor and vaginal delivery was slightly more in PCEA + CI group. This group also consumed more drug per hour. But the difference was not statistically significant (Table 2).

**Table 1:** Patients Characteristics

	Group PCEA n=30	Group PCEA+CI, n=25
• Age in yrs	25±6	24±5
• Weight in Kg	45±16	46±13
• Primi/multi	14/16	11/14
• Gestational age (weeks)	38±1	38±1
• Duration of labor (minutes)	349±78	342±91
• Need for oxytocin augmentation	9(30%)	6(24%)
• Vaginal/caesarean delivery	24/6	21/4
• Pre-block Cx dilatation (median, range)	4(3-6)	4(3-6)
• Neonatal Apgar score at 5 min	8.37±1.0	8.43±0.93

Data are mean ± SD, n(%)

**Table 2:** Study Results

	Group PCEA	Group PCEA +CI	p value
•Mean time for Ist demand bolus (min)	89±17	108±18	< 0.05
•Total volume of bupivacaine consumed(ml)	50±12	55±9	0.079
• Bupivacaine used ml/hr	9±1.2	10±1	0.065
• Mean VAS before block	6.9±0.73	7.7±1.02	0.18
• Mean VAS during labor	1.96±1.08	1.89±1.03	0.32
• Highest VAS (median & range)	6 (0-6)	5 (0-7)	<0.05
• Rescue boluses (no. of patients)	8 (27%)	2 (8%)	<0.05
• MaternalSatisfaction (excellent/good/poor) % of patients	77/17/7	80/16/4	>0.05

The time for first demand bolus dose after initial bolus was significantly longer in PCEA + CI group than in PCEA group. (108±18 vs 89 ± 17 minutes, p<0.05). Analgesia was assessed hourly on a verbal analogue score (VAS). There was no significant difference in mean VAS pain scores in both the groups indicating similar analgesia. The scores were between 0-2 at majority of the assessment times. But more patients (8 vs 2) demanded rescue boluses by the anaesthetist due to distressing pain in PCEA only group (27%) compared to PCEA+CI group (8%) as shown in Table 2. Three patients in PCEA group had more than one episode of distressing pain and 5 had one episode. Two patients who required rescue analgesics had only one such episode in PCEA+CI group. Maternal satisfaction with both the analgesic techniques was high in all patients (Table 2).

## Discussion

Both the groups provided equivalent labor analgesia and maternal satisfaction. The chances of caesarean delivery were also not increased in any group. No difference in the cephalad extent of sensory analgesia, motor block or neonatal Apgar score were observed. Although mean pain scores throughout the labor and delivery were similar in both groups, significantly more patients in PCEA group required supplementary boluses by the anaesthetist due to distressing pain particularly towards the end of first stage of labor. Many previous investigators reported occurrence of breakthrough pain requiring additional rescue analgesia by clinicians^3^,^8^,^9^.

Ocampo et al (2006)^8^ compared PCEA alone to PCEA with 5 or 10 ml/ hour of continuous basal infusion of 0.125% bupivacaine and 2 mcg.ml^−1^ of fentanyl for maintenance of labor analgesia. They reported reduced incidence of breakthrough pain and maximum pain scores in patients receiving PCEA + basal infusion. Maternal satisfaction was also higher in these patients. Similar findings were reported in other studies^3^,^7^,^9^,^10^.

However some authors do not concur with these reports. Boselli et al (2004)^4^ randomly assigned 133 women to received PCEA alone or PCEA with basal infusion at the rate of 3, 6 or 9 ml/hr of 0.1% ropivacaine and 0.5 mcg.ml^−1^ of sufentanil. The verbal pain scores, number of supplemental boluses and maternal satisfaction was comparable in all the four groups. The patients receiving 6 or 9 ml/hour of basal infusion consumed more ropivacaine but without any untoward effect such as dense motor block or higher sensory level or hypotension. They concluded that background in-fusion was unnecessary as it did not improve maternal comfort and satisfaction or obstetrical outcome despite use of more local anaesthetic and opioid but increased the cost of cares. Theonaz et a (2006)^6^ supported their view and recommended against the use of continuous basal infusion along with PCEA.

A potential drawback of PCEA technique is risk of patients receiving higher amount of local anaesthetic leading to high block^12^. Though our patients of PCEA+CI group received slightly higher amount of bupivacaine, higher block was not seen in any patient, an observation common with other reports^5^,^6^,^8^,^13^. Maternal satisfaction was also high and equivalent in all the patients. It was surprising that despite having distressing pain sometimes during labor, satisfaction was high. Experiencing pain of short duration was considered satisfactory by some women and probably this amounted to equal satisfaction in both groups.

PCEA can theoretically adjust for individual pain threshold, allowing patients to use only the analgesic dose needed^1^,^11^,^12^. The results of this study and other studies^3^,^8^,^9^ show that, while providing labor analgesia by PCEAonly, many women experienced breakthrough pain. It has been suggested that this could be due to lock out interval and maximum dose limit restricting the drug delivery, thus questioning the adequacy of only PCEA in parturients^1^. As the labor advances the contractions become more painful requiring more bupivacaine but the dose of bupivacaine remains unchanged due to PCA pump setting. When continuous basal infusion is incorporated with PCEA, this problem is obviated to some extent. Therefore the frequency of break through pain and additional boluses that are required to be given by anaesthetist are reduced. It allowed sustained alleviation of pain^9^,^14^ along with saving of manpower^12^,^14^,^15^

On the basis of our results we found that the technique of PCEA during labor was safe, effective and gave the women a feeling of ‘self control’ on pain. The addition of background infusion to PCEA resulted in more constant pain relief with lesser episodes of distressing pain demanding rescue boluses from anaesthetist. In fact it is difficult to convincingly prove which method is superior to other in a relatively small sample size. Before conclusion we say that each centre should select a technique which is suitable depending upon the availability of anaesthetist, equipment and expectation of parturient as well as obstetrician.
